# Non-invasive liver fibrosis screening on CT images using radiomics

**DOI:** 10.1186/s12880-025-01823-w

**Published:** 2025-07-15

**Authors:** Jay J. Yoo, Khashayar Namdar, Sean Carey, Sandra E. Fischer, Chris McIntosh, Farzad Khalvati, Patrik Rogalla

**Affiliations:** 1 Institute of Medical Science, University of Toronto, 1 King’s College Circle, Toronto, ON M5S 1A8 Canada; 2 Department of Diagnostic Imaging & Interventional Radiology, The Hospital for Sick Children, 555 University Avenue, Toronto, ON M5G 1X8 Canada; 3 Department of Medical Imaging, University of Toronto, 263 McCaul Street, Toronto, ON M5T 1W7 Canada; 4 Department of Computer Science, University of Toronto, 40 St. George Street, Toronto, ON M5S 2E4 Canada; 5 Department of Mechanical and Industrial Engineering, University of Toronto, 5 King’s College Road, Toronto, ON M5S 3G8 Canada; 6 Vector Institute, 661 University Avenue, Toronto, ON M5G 1M1 Canada; 7 Joint Department of Medical Imaging, University of Toronto, Toronto General Hospital, 585 University Avenue, Toronto, ON M5G 2N2 Canada; 8 Department of Medical Biophysics, University of Toronto, 101 College Street, Toronto, ON M5G 1L7 Canada; 9 Techna Institute, University Health Network, 190 Elizabeth Street, Toronto, ON M5G 2C4 Canada; 10 Radiation Medicine Program, Princess Margaret Cancer Centre, 610 University Avenue, Toronto, ON M5G 2C4 Canada; 11 Peter Munk Cardiac Center, University Health Network, 585 University Avenue, Toronto, ON M5G 2N2 Canada; 12 Laboratory Medicine & Pathobiology - Anatomic Pathology, University of Toronto, Toronto General Hospital, 585 University Avenue, Toronto, ON M5G 2N2 Canada

**Keywords:** Machine learning, Liver, Fibrosis, Computed tomography, Radiomics

## Abstract

**Purpose:**

To develop a radiomics machine learning model for detecting liver fibrosis on CT images of the liver.

**Methods:**

With Ethics Board approval, 169 patients (68 women, 101 men; mean age, 51.2 years ± 14.7 [SD]) underwent an ultrasound-guided liver biopsy with simultaneous CT acquisitions without and following intravenous contrast material administration. Radiomic features were extracted from two regions of interest (ROIs) on the CT images, one placed at the biopsy site and another distant from the biopsy site. A development cohort, which was split further into training and validation cohorts across 100 trials, was used to determine the optimal combinations of contrast, normalization, machine learning model, and radiomic features for liver fibrosis detection based on their Area Under the Receiver Operating Characteristic curve (AUC) on the validation cohort. The optimal combinations were then used to develop one final liver fibrosis model which was evaluated on a test cohort.

**Results:**

When averaging the AUC across all combinations, non-contrast enhanced (NC) CT (AUC, 0.6100; 95% CI: 0.5897, 0.6303) outperformed contrast-enhanced CT (AUC, 0.5680; 95% CI: 0.5471, 0.5890). The most effective model was found to be a logistic regression model with input features of maximum, energy, kurtosis, skewness, and small area high gray level emphasis extracted from non-contrast enhanced NC CT normalized using Gamma correction with γ = 1.5 (AUC, 0.7833; 95% CI: 0.7821, 0.7845).

**Conclusions:**

The presented radiomics-based logistic regression model holds promise as a non-invasive detection tool for subclinical, asymptomatic liver fibrosis. The model may serve as an opportunistic liver fibrosis screening tool when operated in the background during routine CT examinations covering liver parenchyma. The final liver fibrosis detection model is made publicly available at: https://github.com/IMICSLab/RadiomicsLiverFibrosisDetection.

## Introduction

Fibrosis is the wound-healing response to liver injury that can lead to cirrhosis and is associated with an increase in liver-related complications in patients with nonalcoholic steatohepatitis (NASH) [1]. Accurate diagnosis in the pre-cirrhotic phase enables early application of preventative and corrective measures to help prevent the progression of the disease. Therefore, it appears desirable to screen the general population for liver NASH-fibrosis since individuals in the early stages of fibrosis are frequently asymptomatic and may exhibit normal transaminase blood levels. Image-guided liver biopsy is the diagnostic reference standard if NASH is suspected, but it is costly, introduces the risk of biopsy-related complications, and demonstrates low intra- and interobserver repeatability [2, 3]. Furthermore, due to the geographic distribution of NASH and hepatic fibrosis, its accuracy suffers from a sampling error ranging between 55% and 75% [4]. These challenges have led to an interest in developing non-invasive modalities for fibrosis detection [5, 6].

Liver fibrosis is commonly staged into the 5-point METAVIR scoring system (F0: no fibrosis, F1: mild fibrosis, F2: moderate fibrosis, F3: severe fibrosis, F4: cirrhosis) [7]. Prior studies have extensively demonstrated and evaluated the value of applying radiomics to CT in detecting fibrosis stages of F2 and above [8–10]. However, using radiomics to differentiate healthy liver (F0) from diseased liver (F1-F4) on CT images is rarely explored. Instead, the prevalent task is to differentiate lower fibrosis stages (F0-F1) from higher fibrosis stages (F2-F4) [11, 12]. Works that do differentiate F0 from F1 aim to perform 5-class classification using all fibrosis stages (F0, F1, F2, F3, F4), rather than specifically optimizing to distinguish healthy liver from diseased liver [13].

Although magnetic resonance imaging (MRI), including MRI elastography, and ultrasound (US) elastography are established imaging modalities allowing for liver fibrosis quantification [14–16], the motivation to explore computed tomography (CT) as a potential opportunistic screening modality is the possibility of seamlessly incorporating detection algorithms into CT examinations clinically indicated for reasons other than diffuse liver disease. Access and costs are often perceived as barriers to using MRI for screening liver fibrosis, particularly in resource-limited settings [17, 18], and US is impacted by operator variability [19–21] and body mass index-challenged patients [22]. An opportunistic screening tool deployed during subclinical non-indicated CT protocols would enable liver effective liver fibrosis without the associated limitations of MRI and US.

We hypothesized that a radiomics-based machine learning (ML) model, trained using liver CT images (non-contrast and post-contrast) with biopsy as the reference standard, would allow for the differentiation of healthy livers (F0) from fibrotic livers of any stage (F1-F4).

## Methods

### Patients and imaging

This prospective study was approved by the institutional Research Ethics Board (REB) of the University Health Network (UHN) (Toronto, Canada), and written informed consent was obtained from all patients. Between October 2019 to April 2021, 202 patients met the inclusion criteria of having a clinical indication for random liver biopsy and being 18 years of age or older, 2 of which declined consent, resulting in 200 consecutive patients enrolled in the trial.

Accepted clinical indications were to rule out liver fibrosis due to autoimmune hepatitis (AIH), hepatitis C, and elevated enzymes of unknown clinical cause. Ultrasound was used to guide the biopsy needle to a random location in the inferior right liver lobe (preferably segment 5/6) using a 17 gauge coaxial needle, and once the needle was in the desired location, two 8 cm volumetric CTs were obtained over the liver (Aquilion ONE Genesis, Canon Medical Systems, Otawara, Japan) before (NC) and 70 s after injection of intravenous contrast material (CE). Images were acquired using dual energy at 80kVp and 135kVp, which were used to reconstruct 120kVp equivalent images using hybrid-iterative reconstruction (AIDR-3D, Canon Medical Systems). Thin slice axial images were reconstructed and resampled to 0.5 × 0.5 × 0.5 mm voxel size using B-spline to standardize the voxel sizes. Following the CT, an 18 gauge BioPince needle was inserted through the coaxial needle, and a 3-cm full-size core was harvested. The specimen handling and patient observation were done according to standard-of-care practices in our institution.

**Fig. 1:**
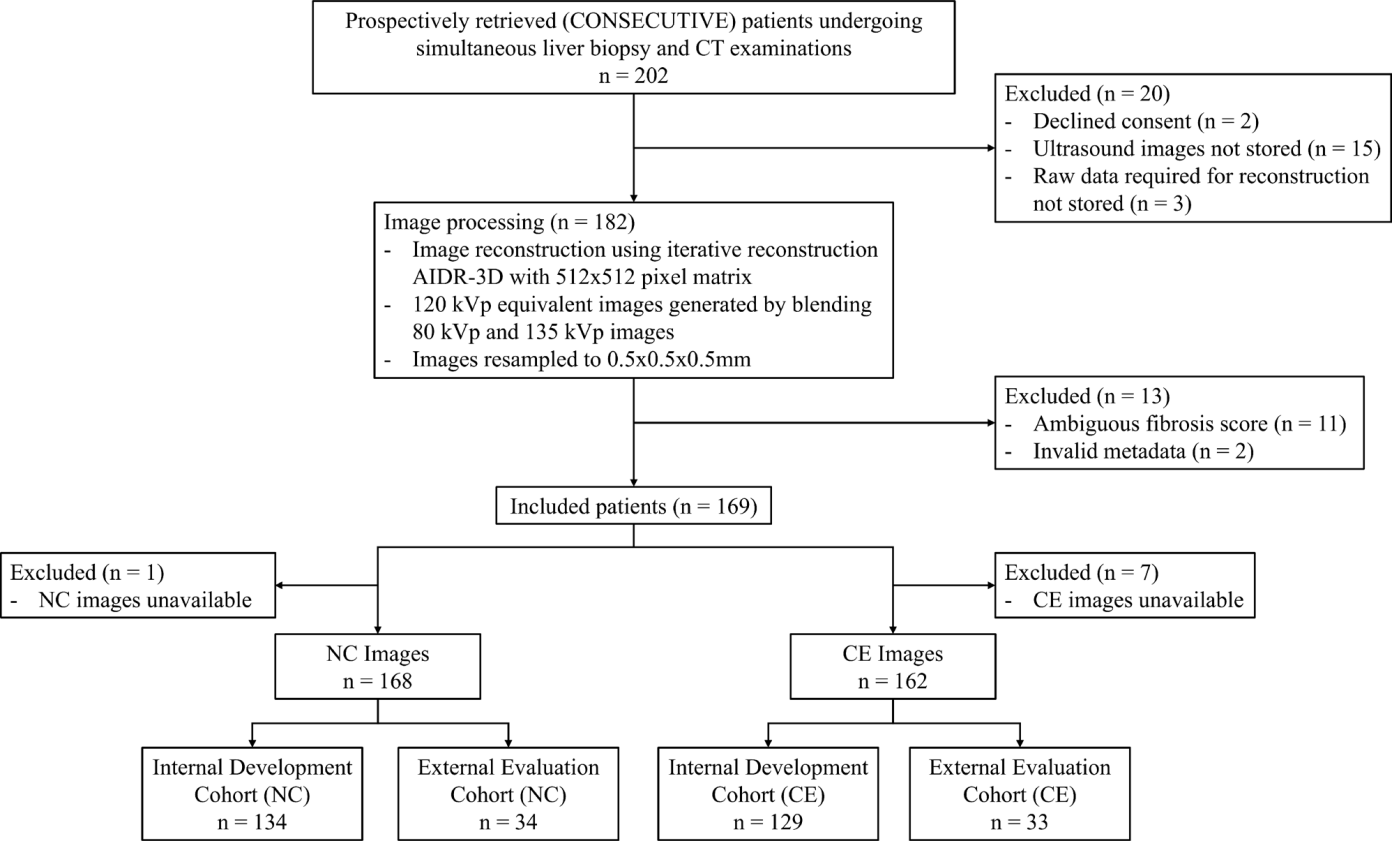
Flowchart of patient and image eligibility for this study

The biopsy specimens were clinically reported by subspecialized liver pathologists and then used to score each patient’s fibrosis stage on a 5-point scale from F0 to F4. Patient biopsies that could not be confidently scored in one of the categories were excluded from the analysis. 15 patients did not have US images stored in PACS due to a technical connectivity issue, 3 patients did not have the raw data required for reconstruction stored, and images from 2 patients were not included due to having invalid metadata. A total of 169 patients were included in the final analysis. 7 patients did not have CE CT acquired because they did not consent to the use of intravenous contrast material, and one patient only had a CE CT due to the NC CT becoming corrupted, resulting in 168 NC CTs and 162 CE CTs from 169 patients. A flowchart of patient eligibility is presented in Fig. 1. The patient demographics and the number of patients at each fibrosis stage are summarized in Table 1.

**Table 1:** Baseline demographics and clinical characteristics of included patients (*n* = 169)

Characteristic	Total (*n* = 169)	Development cohort		Test cohort	
		NC (*n* = 134)	CE (*n* = 129)	NC (*n* = 34)	CE (*n* = 33)
Mean Age ± Standard Deviation	51.19 ± 14.70	51.66 ± 14.90	50.72 ± 15.38	49.06 ± 14.04	51.79 ± 12.12
Number of Men	101	75	76	25	22
Number of Women	68	59	53	9	11
Number of Patients with F0	64	52	52	12	10
Number of Patients with F1	33	25	24	8	8
Number of Patients with F2	28	21	22	7	6
Number of Patients with F3	25	21	19	4	5
Number of Patients with F4	19	15	12	3	4

### Data Preparation

The fibrosis stages were binarized into F0 and F1-F4, and these binary labels were used as the reference standard for this study, where F0 represents healthy livers and F1-F4 represents fibrotic livers of any stage. The attenuation values for all CTs were thresholded as previously proposed [23]. NC CTs were thresholded to a range of 0 HU to 100 HU, and the attenuation values for CE CTs were thresholded to a range of -10 HU to 200 HU to account for potential minimal density differences of individual voxels due to higher image noise and the increased density of liver parenchyma following contrast material injection. The thresholding served to prevent relevant voxels from being reduced to insignificant attenuation values during normalization due to outlier values outside of the liver.

The following settings were investigated: Contrast: [NC, CE], Normalization: [None, Histogram Equalization, Min-max, Z-score, Gamma correction with γ = 0.5 (Gamma-0.5), Gamma correction with γ = 1.5 (Gamma-1.5)], Machine learning model type: [Logistic regression classifier, Random forest classifier, Support vector machines (SVM), Linear models with stochastic gradient descent (SGD) training], and Feature selection method: [None, Principal component analysis (PCA), Boruta, least absolute shrinkage and selection operator (LASSO) regression]. We evaluated every combination of these settings, which we refer to as configurations, totaling 1728 configurations.

### ROI placement and radiomics extraction

**Fig. 2:**
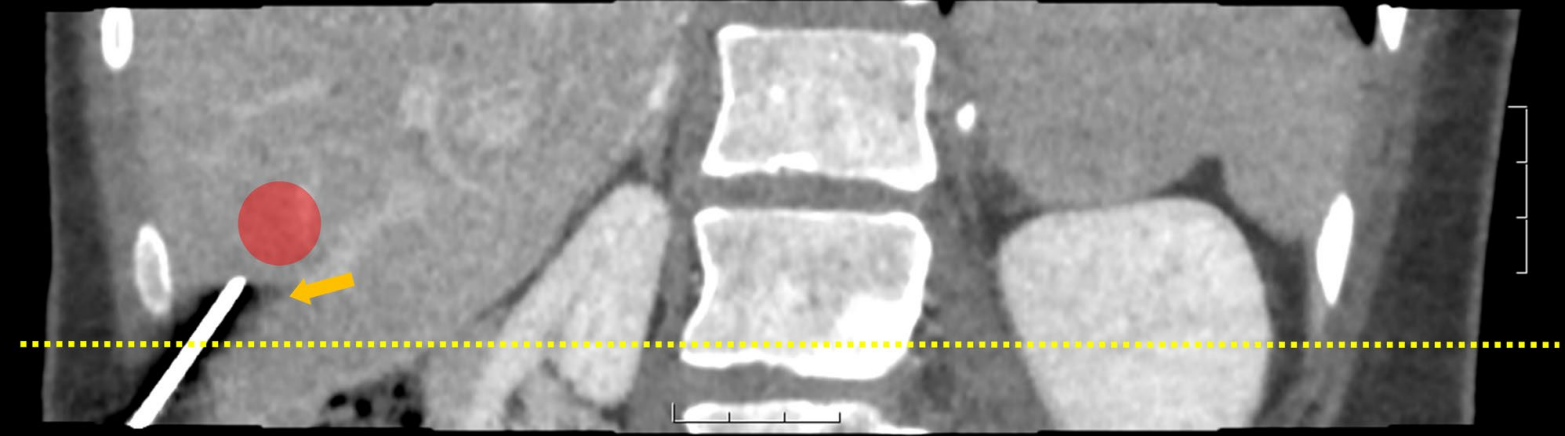
Multiplanar reconstruction view of the liver with an example ROI in red and the needle visualized, as indicated by the yellow arrow. The visualized ROI in the figure does not represent the ROI used to extract radiomic features. The visualized ROI is used to demonstrate how the ROI avoids the artifacts from the needle

The first set of ROIs was placed with their center 2.5 cm distal to the biopsy needle to ensure this set of ROIs was consistent with the corresponding area’s tissue sampling. This set of ROIs is referred to as biopsy-based ROIs, had a radius of 1.5 cm, and served to eliminate a potential sampling error associated with the heterogeneous distribution of fibrosis in the liver. Placing the biopsy-based ROIs 2.5 cm distal to the biopsy needle ensured that the ROIs would not capture streak artifacts on the images caused by the needle. Figure 2 demonstrates how the ROIs avoid streak artifacts. Figure 2 also shows the direction of CT scanning and the subsequent artifact that is avoided by angling the needle. The artifacts occur in the direction of the axial slices only, and artifacts are absent at the tip of the needle.

Another set of 1.5 cm radius ROIs was placed a minimum of 3 cm away from the centers of the biopsy-based ROIs. These ROIs were placed manually in a randomly chosen location with preference in the left liver lobe. This set of ROIs is referred to as non-biopsy ROIs and served to evaluate the ability of radiomics models to detect fibrosis in liver regions outside the biopsy-based ROIs. Each non-biopsy ROI shared the same ground truth as the biopsy-based ROI from the same CT volume.

**Fig. 3:**
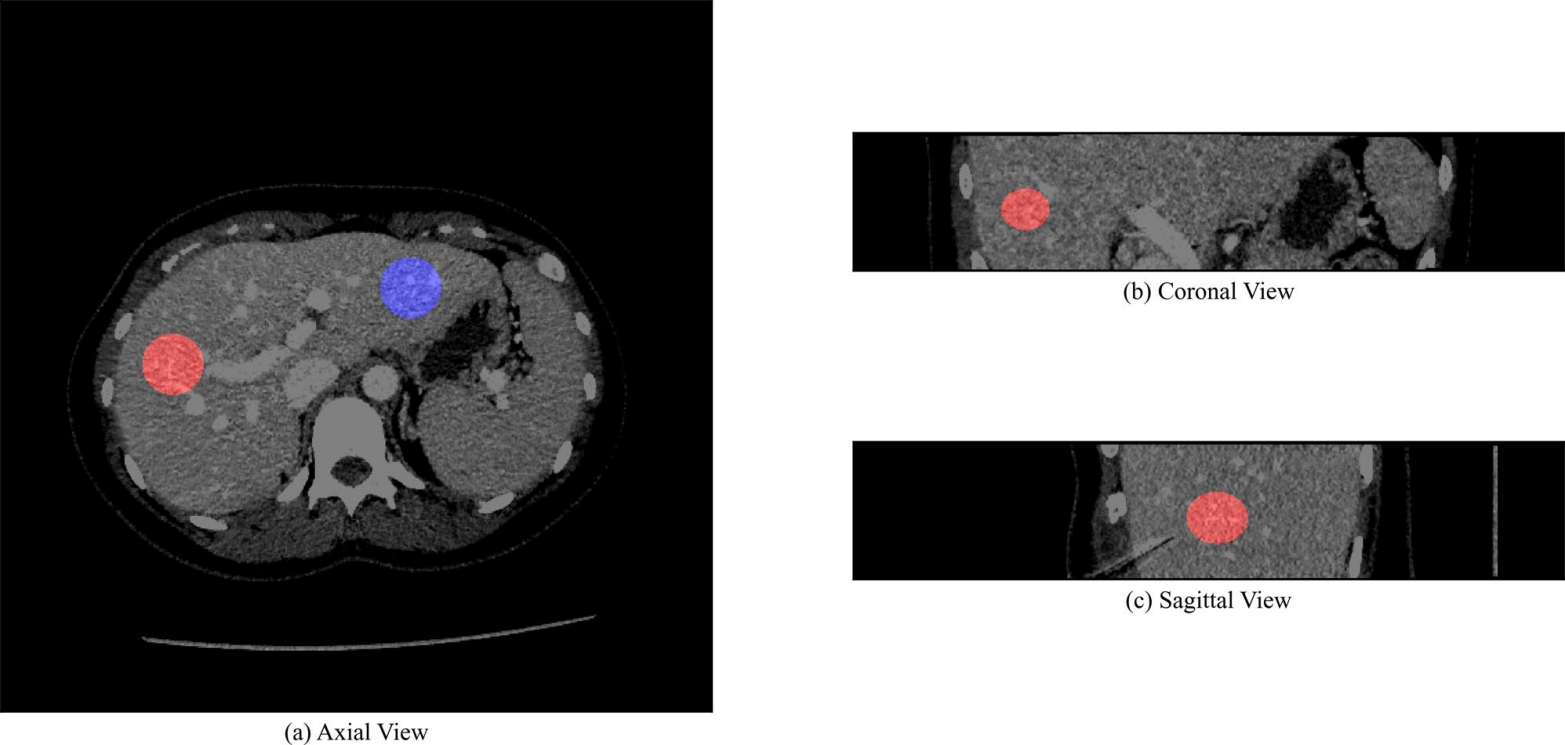
Example CT image with axial (**a**), coronal (**b**), and sagittal (**c**) views. The biopsy-based ROI is visualized in red and the non-biopsy ROI is visualized in blue. The biopsy needle is visible in the sagittal view

Any ROI that included tissue outside of the liver was shifted such that the ROI only contained liver parenchyma, while avoiding larger hepatic vessels (hepatic veins, portal veins). Each shift was a maximum of 3 cm along any axis. An example CT image with the biopsy-based and non-biopsy ROIs and the biopsy needle visualized is presented in Fig. 3. The ROIs were placed and shifted by one human reader. The final position of the ROIs was then verified by a subspecialized abdominal radiologist (PR).

After ROI placement, we used PyRadiomics 3.1.0 [24] to extract 1725 radiomic features from biopsy-based and non-biopsy ROIs. Discretization method parameters were set to their default values as defined in PyRadiomics 3.1.0, all image types were enabled during radiomic feature extraction, and all other PyRadiomics parameters remained at their default values. The images were first thresholded and then normalized prior to the radiomics extraction. All extracted radiomic features were included except for shape features. Shape features were excluded because they lack differentiative power between patients due to the ROIs being the same shape across all patients.

### Experimental design

**Fig. 4:**
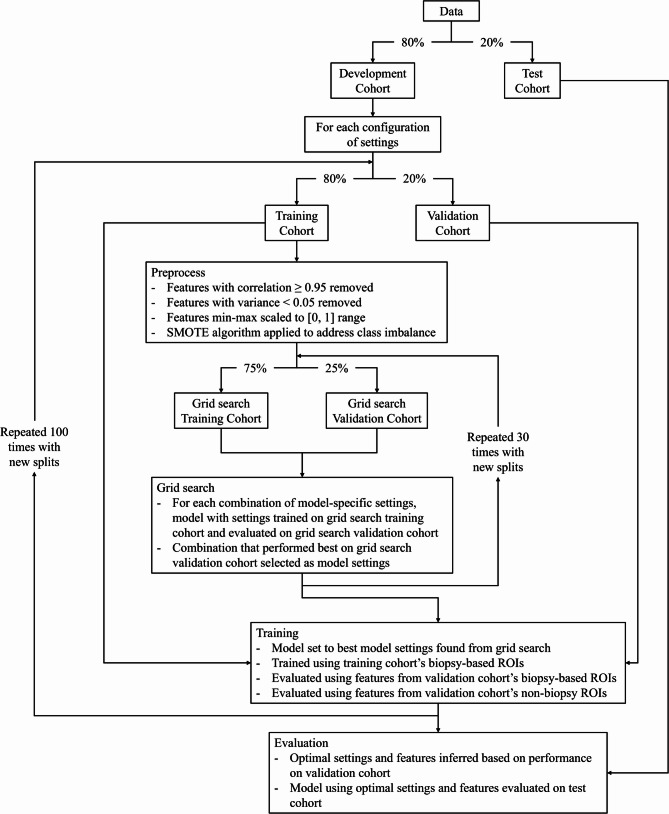
Flowchart of experimental design. Percentages in arrows represent percentage of data in each data split

Figure 4 presents a flowchart of the experimentation design. We first designated 20% of the NC and CE CTs as hold-out test data referred to as the test cohort. For the remaining data, referred to as the development cohort, 100 experiments were run for each configuration of the 1728 configurations as inspired by Liu et al. [25] and Namdar et al. [26]. This loop of 100 experiments is used to determine the configuration of settings most suited for liver fibrosis detection. Each experiment set a training cohort and a validation cohort using a different random 80/20 of the development cohort to increase confidence that the observed performance generalized to unseen data. The features of each split were individually processed by removing features with a correlation greater than 0.95 with another feature or a variance less than 0.05, before being min-max normalized to range [0, 1].

The development cohort included 81 NC CTs and 76 CE CTs with fibrosis, as well as 53 NC CTs and 53 CE CTs without fibrosis. The test cohort included 23 NC CTs and 24 CE CTs with fibrosis, as well as 11 NC CTs and 9 CE CTs without fibrosis. Synthetic Minority Oversampling Technique (SMOTE) algorithm [27] was applied to the training data to address the class imbalances.

During each of the experiments performed on the development cohort, the ML model-specific hyperparameters, listed in Table 2, were determined by performing a grid search of the hyperparameters. The ML model-specific hyperparameters are the hyperparameters that are specific to each of the different ML model settings that were explored in this study and are based on their implementations in the scikit-learn Python library. The grid search was done by training the model using each combination of ML model-specific hyperparameters on 75% of the training cohort and then evaluating the area under the receiver operating characteristic curve (AUC) of the model on the remainder of the training cohort. Each combination of ML-model specific hyperparameters was evaluated 30 times using different 75/25 splits of the training cohort, and the combination yielding the greatest average AUC was selected for the current experiment. Any hyperparameters not mentioned in Table 2 were set to their default value as defined in the scikit-learn library.

**Table 2:** Model-specific settings and their candidate values

Model Type	Hyperparameter	Candidate Values
Logistic Regression	Optimization Solver	L-BFGS-B
		Newton-CG
		Stochastic Average Gradient (SAG)
		Stochastic Average Gradient Ascent (SAGA)
	Inverse Regularization Strength	0.5
		1.0
		1.5
Random Forest	Number of Estimators	50
		100
		200
	Maximum Number of Features	Auto
		Square Root
	Maximum Depth	All
		5
		100
SVM	Kernel	Linear
		Polynomial (degree 3)
		Radial Basis Function (RBF)
	Inverse L2 Squared Regularization Strength	0.5
		1.0
		1.5
Linear Model with SGD	L2 Regularization Strength	0.001
		0.0001
		0.00001
	Loss Function	Logistic Regression
		Modified Huber Loss

Once the model-specific hyperparameters were selected, they were used with the current configuration to train the model using the training cohort and were subsequently evaluated on the validation cohort. The model was evaluated on both the features from the biopsy-based ROIs and the non-biopsy ROIs of the validation cohort, forming two sets of validation results. The features from the non-biopsy ROIs were only used for evaluation and were not used to train the models. In clinical settings, the models would be applied to multiple randomly selected non-biopsy ROIs. The average prediction across the ROIs would then determine the final prediction.

The experiments were then analyzed to determine the most effective radiomic features for liver fibrosis detection. These features were selected by observing the most common features used by the five configurations with the greatest mean validation AUCs on the biopsy-based ROIs and the five configurations with the greatest mean validation AUCs on the non-biopsy ROIs. The features for the biopsy-based ROIs reflected the ROIs used to train the model and the features for the non-biopsy ROIs reflected the potentially relevant features in a non-invasive setting where biopsy-based ROIs would not be available. The selected features were used to train models that we will refer to as simple due to the lower number of input features. The hyperparameters used for the simple models were the hyperparameters that yielded the greatest validation AUC when evaluating the different configurations of hyperparameters. The validation cohort was used as it allowed for the most effective configurations to be determined for use in the final liver fibrosis detection model, which was evaluated on the test cohort.

As a baseline, we evaluated a model based on the work by Hirano et al. [28] which differentiated healthy livers (F0) from fibrotic livers (F1-F4) on NC CT images as was done in our study. This method passed the following three features extracted from 5 manually selected cubic ROIs to a logistic regression model with L2 regularization: 2D wavelet decomposition feature, standard deviation of variance filter, and mean CT intensity.

AUC, sensitivity, and specificity were used to evaluate models on the test cohort. p-values for the AUC results were also calculated using the two-tailed paired Delong’s test with α = 0.05 and compared each model to the simple model trained using the non-biopsy feature set.

## Results

### Configuration selection

To determine the optimal combinations of settings to use when developing the final liver fibrosis detection model, the configurations were sorted based on their mean validation AUC and the five configurations with the greatest validation AUC on the biopsy-based ROIs and the non-biopsy ROIs were observed. These results are presented in Table 3.

**Table 3:** AUCs for the five best configurations evaluated on validation cohorts of biopsy-based and non-biopsy ROIs

Test ROI	Settings				AUC
	Normalization	Feature Selection	Model	Contrast	
Biopsy-based ROIs	Gamma-1.5	Boruta	SGD	NC	0.7390; 95% CI: [0.7229, 0.7551]
	Gamma-1.5	Boruta	Logistic Regression	NC	0.7356; 95% CI: [0.7188, 0.7524]
	Min-Max	Boruta	SGD	NC	0.7329; 95% CI: [0.7153, 0.7504]
	Gamma-1.5	LASSO	Logistic Regression	NC	0.7302; 95% CI: [0.7135, 0.7470]
	Min-Max	Boruta	Logistic Regression	NC	0.7256; 95% CI: [0.7071, 0.7440]
Non-biopsy ROIs	Gamma-1.5	Boruta	Logistic Regression	NC	0.6726; 95% CI: [0.6545, 0.6906]
	None	Boruta	SGD	NC	0.6704; 95% CI: [0.6515, 0.6892]
	None	Boruta	Logistic Regression	NC	0.6701; 95% CI: [0.6505, 0.6896]
	Gamma-1.5	Boruta	SGD	NC	0.6660; 95% CI: [0.6483, 0.6838]
	Gamma-1.5	Boruta	SVM	NC	0.6592; 95% CI: [0.6404, 0.6780]

The configurations with the greatest mean validation AUCs on the biopsy-based ROIs and non-biopsy ROIs both had Gamma-1.5 normalization, Boruta feature selection, and used NC images. However, the configurations had different model settings. Between linear models with SGD training and logistic regression, we concluded that logistic regression is more suitable for liver fibrosis detection because it had the greater mean validation AUC on the non-biopsy ROIs, which are more indicative of how the model will perform as a liver fibrosis screening tool than the biopsy-based ROIs. Therefore, Gamma-1.5 normalization, Boruta feature selection, logistic regression on non-contrast CT images were selected as the configuration to be used to train the final liver fibrosis detection models.

### Radiomic feature selection

**Fig. 5:**
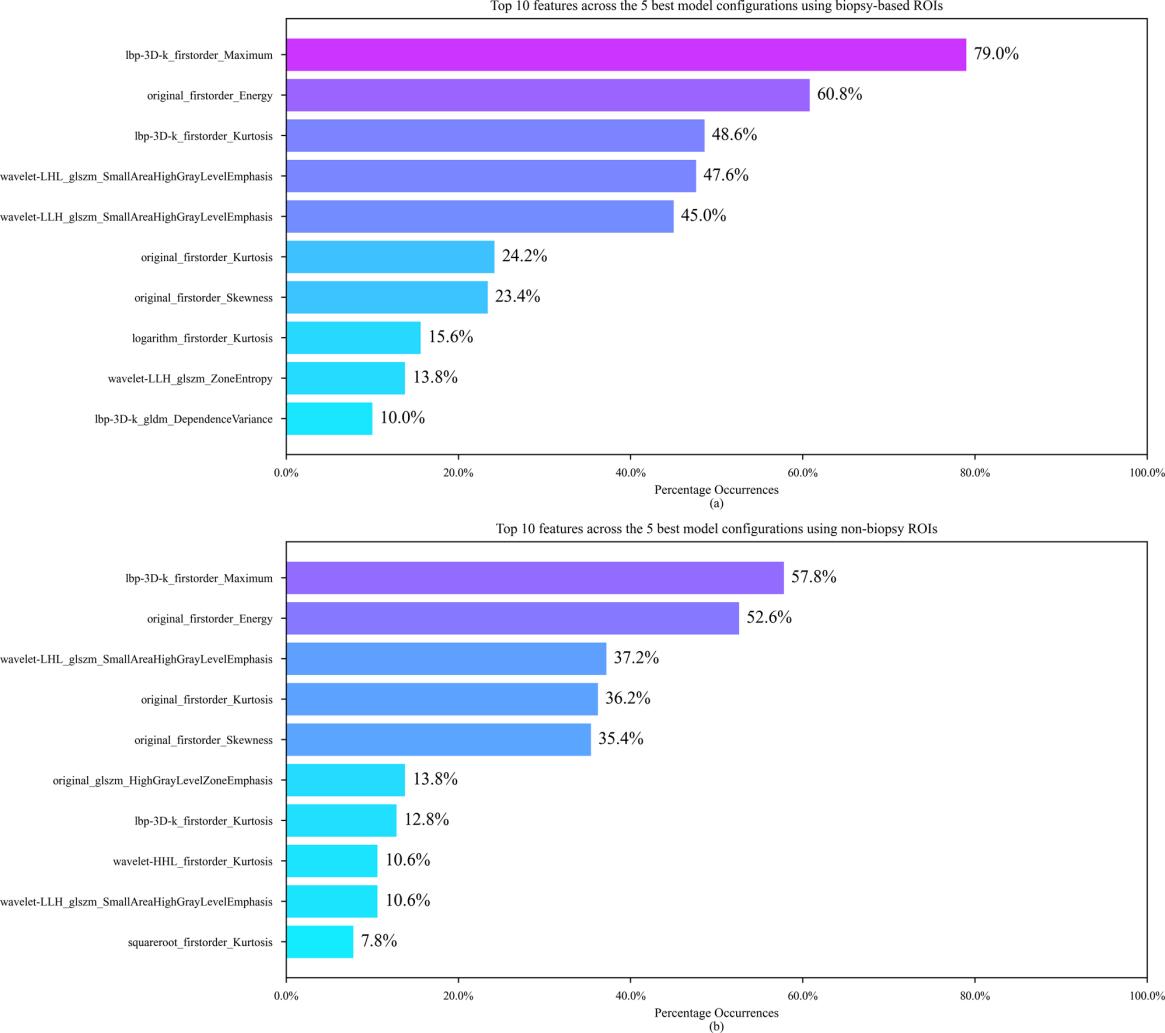
Top 10 features across the 5 best configurations for each set of ROIs. (**a**) Top 10 features across the 5 best configurations using biopsy-based ROIs. (**b**) Top 10 features across the 5 best configurations using non-biopsy ROIs

To determine the radiomic features that best differentiate liver fibrosis, we observed the most common features amongst the 10 configurations that were presented in Table 3. The 10 most common features across the five configurations with the greatest mean validation AUCs on the biopsy-based ROIs and the the 10 most common features across the five configurations with the greatest mean validation AUCs on the non-biopsy ROIs are sorted and presented in Fig. 5. It can be observed that for both of these feature rankings, the top 5 features were significantly more common than the other features.

Based on these observations, the 5 most common features across the 5 highest performing biopsy-based configurations, shown in Fig. 5 (a), were grouped and named the biopsy-based feature set (lbp-3D-k_firstorder_Maximum, original_firstorder_Energy, lbp-3D-k_firstorder_Kurtosis, wavelet-LHL_glszm_SmallAreaHighGrayLevelEmphasis, and wavelet-LLH_glszm_SmallAreaHighGrayLevelEmphasis), and the 5 most common features across the 5 highest performing non-biopsy configurations, shown in Fig. 5 (b), were grouped and named the non-biopsy feature set (lbp-3D-k_firstorder_Maximum, original_firstorder_Energy, wavelet-LHL_glszm_SmallAreaHighGrayLevelEmphasis, original_firstorder_Kurtosis, and original_firstorder_Skewness).

**Fig. 6:**
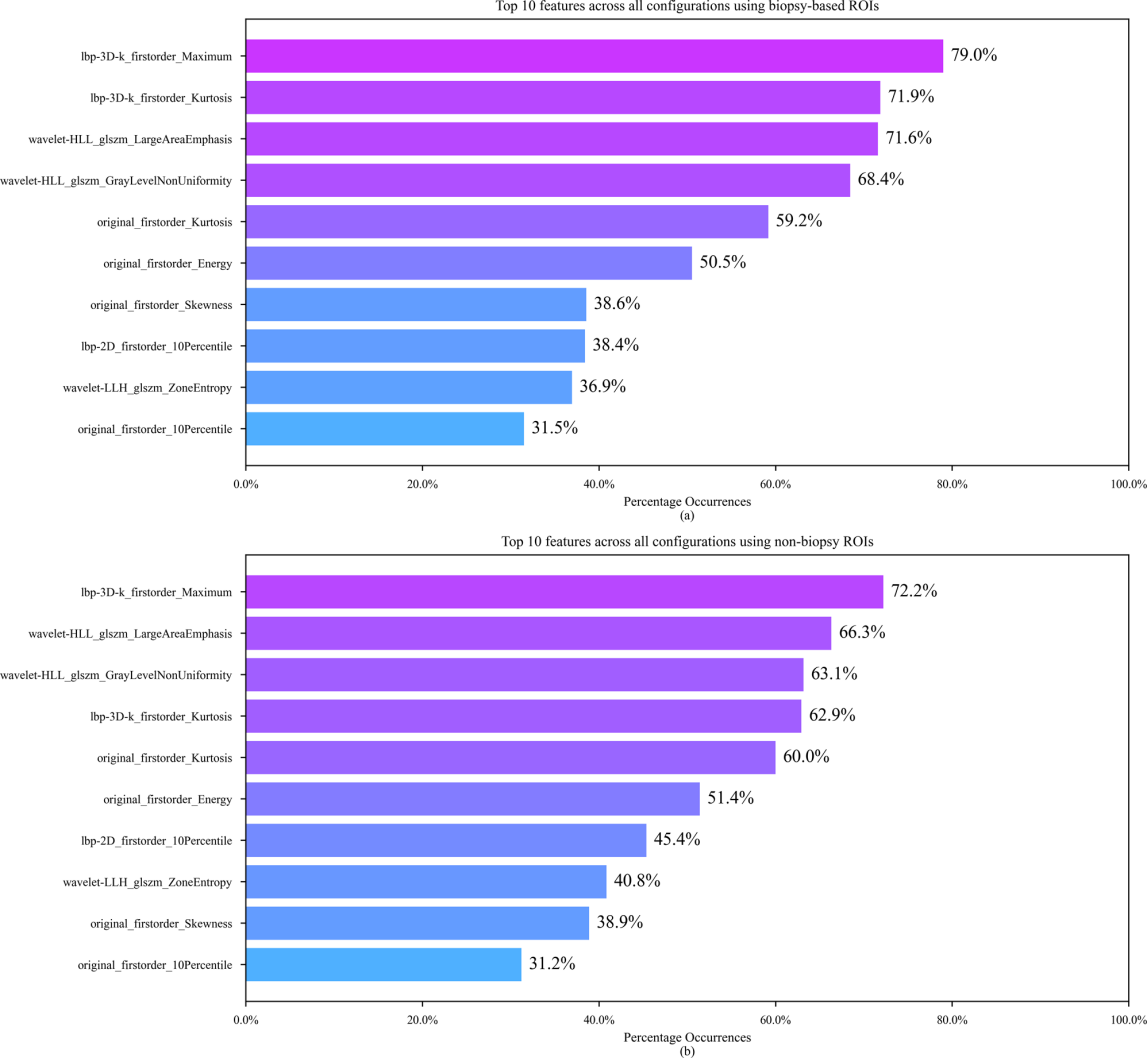
Top 10 features across all configurations for each set of ROIs. (**a**) Top 10 features across all configurations using biopsy-based ROIs. (**b**) Top 10 features across all configurations using non-biopsy ROIs

For completeness, we also present the top 10 features across all configurations and experiments in Fig. 6. The lbp-3D-k_firstorder_Maximum, original_firstorder_Energy, and lbp-3D-k_firstorder_Kurtosis features from the biopsy-based feature set are also among the top 10 features across all experiments using biopsy-based ROIs as shown in Fig. 6 (a). From the non-biopsy feature set, lbp-3D-k_firstorder_Maximum, original_firstorder_Energy, original_firstorder_Kurtosis, and original_firstorder_Skewness appear in the top 10 features across all experiments using non-biopsy ROIs as shown in Fig. 6 (b).

### Final liver fibrosis detection model performance

We used the biopsy-based and non-biopsy feature sets to train two simple models. These models used radiomic features extracted from NC CT images normalized using Gamma-1.5 normalization were used to train logistic regression models, which correspond to the configuration selected to train the final liver fibrosis detection model. As a baseline, we evaluated the model proposed by Hirano et al. [28].

All of these models were trained on the development cohort and evaluated on both the biopsy-based and non-biopsy ROIs from the test cohort to determine the most effective liver fibrosis detection model. It is important to clarify that the terms biopsy-based feature set and non-biopsy feature set do not refer to the ROIs that they are extracted from. Rather, these feature sets are combinations of features that were determined using the biopsy-based ROIs and non-biopsy ROIs from the validation cohort. Therefore, when evaluating the performance of a simple model on biopsy-based ROIs using the non-biopsy feature set as the input features, all the features input to the model are extracted from the biopsy-based ROIs. Accordingly, when evaluating the performance of a simple model on non-biopsy ROIs using the biopsy-based feature set as the input features, all the features input to the model are extracted from non-biopsy ROIs.

**Fig. 7:**
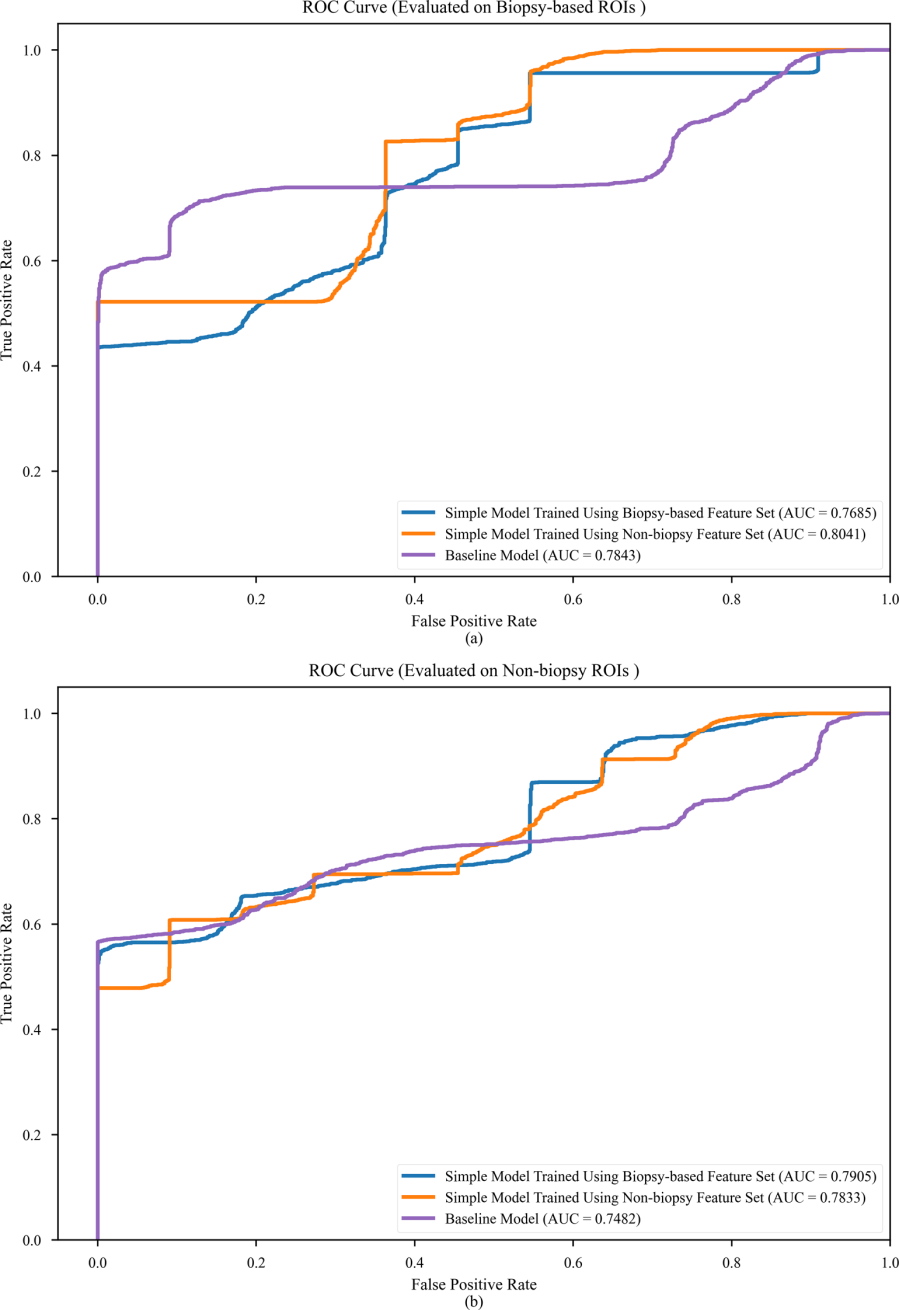
ROC curves for the final liver fibrosis detection models evaluated on the test cohort. ROC curves for simple models and baseline model as evaluated on biopsy-based ROIs (**a**) and non-biopsy ROIs (**b**)

Table 4 presents the AUCs and the corresponding p-values, as well as the sensitivity and specificity metrics for these models as evaluated on the test cohort. Receiver operating characteristic (ROC) curves for the models presented in Table 4 can be found in Fig. 7. The simple model trained on the biopsy-based feature set (AUC on biopsy-based ROIs: 0.7685, AUC on non-biopsy ROIs: 0.7905) outperformed the baseline model in test AUC on the non-biopsy ROIs, while the the simple model trained on the non-biopsy feature set (AUC on biopsy-based ROIs: 0.8041, AUC on non-biopsy ROIs: 0.7833) outperformed the baseline model in test AUC on both the biopsy-based and non-biopsy ROIs. It should be noted that the baseline model outperformed the biopsy-based simple model on biopsy-based ROIs. While the simple model trained on the biopsy-based feature set slightly outperformed the model trained on the non-biopsy feature set on non-biopsy ROIs, the latter model significantly outperformed the former model when evaluated on biopsy-based ROIs. As such, we determined the simple model trained on the non-biopsy feature to be the most effective for liver fibrosis detection.

**Table 4:** Comparison of final liver fibrosis detection models evaluated on the test cohort

Model	Biopsy-based ROIs				Non-biopsy ROIs			
	AUC		Sensitivity	Specificity	AUC		Sensitivity	Specificity
	Mean	*p*-value			Mean	*p*-value		
Simple Model Using Biopsy-based Feature Set	0.7685; 95% CI: [0.7677, 0.7693]	9.564e-2	0.6144; 95% CI: [0.6091, 0.6196]	0.6446; 95% CI: [0.6378, 0.6513]	0.7905; 95% CI: [0.7893, 0.7916]	6.213e-10	0.5661; 95% CI: [0.5649, 0.5673]	0.8936; 95% CI: [0.8869, 0.9003]
Simple Model Using Non-biopsy Feature Set	0.8041; 95% CI: [0.8032, 0.8049]	1.199e-3	0.5217; 95% CI: [0.5217, 0.5217]	0.7227; 95% CI: [0.7181, 0.7274]	0.7833; 95% CI: [0.7821, 0.7845]	1.463e-7	0.5283; 95% CI: [0.5231, 0.5334]	0.9091; 95% CI: [0.9091, 0.9091]
Baseline Model	0.7843; 95% CI: [0.7824, 0.7862]	-	0.6035; 95% CI: [0.6007, 0.60625]	0.9400; 95% CI: [0.9316, 0.9484]	0.7482; 95% CI: [0.7457, 0.7508]	-	0.5657; 95% CI: [0.5648, 0.5665]	1.000; 95% CI: [1.000, 1.000]

AUC, p-value, sensitivity, and specificity of simple models compared to models trained using all features and the baseline model, evaluated on both sets of ROIs from the test cohort. The p-values were computed using the two-tailed paired Delong’s test with α = 0.05

### Robustness to region of interest volume confoundment

Welch et al. [29] found that features from PyRadiomics that are dependent on image attenuation such as energy can effectively serve as surrogates for delineated tumor volume, even when shape features such as volume are not meant to be considered. To ensure that the findings of this work are independent of the ROI volume, a model was trained using the MeshVolume feature, the primary volume metric for PyRadiomics, and another model was trained using the pixel spacing values of each image. Both models were trained using NC images normalized using Gamma-1.5 normalization as was done for the simple models. No feature selection was used to ensure that the volume-related features were considered by the models. The models trained using MeshVolume were trained using a logistic regression model while the model trained using pixel spacing values were trained using logistic regression models and random forest models. The pixel spacing serves as an effective measure of ROI volume as all ROIs are spheres with 1.5 cm radii. Thus, the differences between the volumes of ROIs between images can be measured using the pixel spacing as they impact how many voxels constitute the ROI. The performances of these models are presented in Table 5.

**Table 5:** Mean test AUC of volume dependent models

Model	Biopsy-based ROIs	Non-biopsy ROIs
Mesh Volume Models	0.4980; 95% CI: [0.4980, 0.4980]	0.5968; 95% CI: [0.5968, 0.5968]
Pixel Spacing Models	0.5524; 95% CI: [0.5508, 0.5540]	0.5524; 95% CI: [0.5508, 0.5540]

## Discussion

Our study of ML-based liver fibrosis detection on CT images using radiomic features demonstrated that using logistic regression classifiers on non-contrast CT images normalized using Gamma-1.5 presents potential value for screening subclinical liver fibrosis that would otherwise remain undetected. A significant strength of our study is the use of ROIs that are spatially synchronized with the biopsy location and temporally synchronized with the image measurement. We have made the presented model publicly available at https://github.com/IMICSLab/RadiomicsLiverFibrosisDetection.

Our model achieved AUCs of 0.8041 and 0.7833 on biopsy-based and non-biopsy ROIs, respectively, surpassing the AUC of 0.78 reported by Lubner et al. in their study [30]. In addition, our work targets liver fibrosis detection whereas existing works on radiomics applied to CT primarily investigated differentiating F0-F1 from F2-F4 [8–12]. The only other work that we are aware of that specifically investigates liver fibrosis detection is the work by Hirano et al. [28]. We found that our proposed model outperformed the model proposed by Hirano et al. when trained and evaluated on our dataset.

While the performance of our proposed model on the biopsy-based and non-biopsy ROIs are both valuable in interpreting the effectiveness of the model, in clinical practice the performance of the model on non-biopsy ROIs has greater importance than the performance of the model on biopsy-based ROIs. This is because during non-invasive screening, the location of biopsy-based ROIs will not exist, and random ROI sampling will be required to use the proposed model. The similar performance of our proposed model on the biopsy-based and non-biopsy ROIs is indicative of the applicability of the proposed model when biopsy-based ROIs are not present.

MRI has been shown to be superior to CT for detecting liver fibrosis [31, 32]. However, despite MRI’s potential advantages, CT and MRI serve distinct purposes. Extracting radiomic features from CT images, which has been proven to be effective in differentiating pancreatic ductal adenocarcinoma (PDAC) from pancreatic adenosquamous carcinoma [33] and staging [34] PDAC, presents an opportunity to screen patients for onset liver fibrosis. Detecting liver fibrosis during CT examinations, which are often performed for clinical reasons unrelated to liver diseases, allows for opportunistic detection of early, subclinical fibrosis, potentially enabling earlier therapeutic interventions, from lifestyle changes to novel treatments options.

To illustrate the applicability of the proposed model and its potential contribution to the diagnosis of liver fibrosis, we envision the model to silently evaluate CT images from patients undergoing CT imaging. The model could use multiple randomly selected ROIs within the liver to increase the robustness of the prediction. If the model predicts a patient to have liver fibrosis, the patient can then be recommended to undergo MRI or US imaging to stage the liver fibrosis and begin treatment if clinically appropriate. This process would increase the number of patients identified with early onset liver fibrosis without the limited access or costs associated with MRI or the variability associated with US.

Because of the geographical distribution of fibrotic liver disease, we used ROIs distal to the biopsy needle due to concern that designating ROIs significantly distant from the biopsy needle might result in an ROI that captured an area with a different fibrosis stage than the fibrosis stage specified from the biopsy. While this issue is addressed by also using random non-biopsy ROIs, the use of biopsy-based ROIs in a study on non-invasive liver fibrosis detection can introduce unexpected bias to the model, despite the benefits that come with using ROIs spatially and temporally synchronized with the ground truth of specimens’ pathology.

All patients included in our study had a clinical indication for a random liver biopsy, which may have introduced a selection bias. However, performing a liver biopsy in patients without a clinical indication would simply be prohibitive. Furthermore, the ROIs were placed based on a single human reader, introducing a risk that another human reader would have placed slightly different ROIs, thereby impacting the radiomic features. Another limitation of this study is that, while Gamma correction with γ = 1.5 outperformed γ = 0.5, higher γ values were not explored due to computational constraints. It is, therefore, possible that higher values could further improve the classification performance. In addition, our proposed model cannot be used in cases where only CE images are available, as it was trained on NC images. The choice to evaluate ROIs rather than the entire liver parenchyma can be considered another limitation of this study. However, segmenting the liver on NC and low-dose CT may become challenging, particularly in patients with little intra-abdominal fat. Nevertheless, using the entire liver instead of ROIs can be considered potential future research.

## Conclusion

We present a model for the potential use as an opportunistic, non-invasive liver fibrosis screening tool on CT images acquired for any clinical reason. Our model could run in the background to detect unexpected, clinically silent fibrosis. The tool would apply the model to numerous random liver ROIs extracted from NC CT images. The predictions would then be aggregated to determine an overall likelihood for the patient to have liver fibrosis. Using multiple ROIs may remove the need to sample the ROIs at a particular location and mitigate the risk of the geographic distribution of the disease skewing the prediction. Patients detected by the model to have liver fibrosis can then be recommended for further evaluation using alternative imaging tests and further clinical workup, potentially developing an appropriate prevention and treatment plan.

## Data Availability

The datasets generated and/or analyzed during the current study are available from the corresponding author on reasonable request pending the approval of the institution(s) and trial/study investigators who contributed to the dataset.

## Abbreviations

AIH: Autoimmune hepatitis
AUC: Area under the receiver operating characteristic curve
CE: Contrast-enhanced
CT: Computed tomography
Gamma-0.5: Gamma correction with γ = 0.5
Gamma-1.5: Gamma correction with γ = 1.5
LASSO: Least absolute shrinkage and selection operator
ML: Machine learning
MRI: Magnetic resonance imaging
NASH: Nonalcoholic steatohepatitis
NC: Non-contrast
PCA: Principal component analysis
REB: Research ethics board
ROC: Receiver operating characteristic
SGD: Stochastic gradient descent
SMOTE: Synthetic minority oversampling technique
SVM: Support vector machine
US: Ultrasound
